# The dynamics of monocytes and microglia in Alzheimer’s disease

**DOI:** 10.1186/s13195-015-0125-2

**Published:** 2015-04-15

**Authors:** Peter Thériault, Ayman ElAli, Serge Rivest

**Affiliations:** Department of Molecular Medicine, Neuroscience Laboratory, CHU de Québec Research Center (CHUL), Faculty of Medicine, Laval University, 2705 Laurier Boulevard, Quebec City, QC G1V 4G2 Canada

## Abstract

Alzheimer’s disease (AD) is the most common neurodegenerative disorder affecting older people worldwide. It is a progressive disorder mainly characterized by the presence of amyloid-beta (Aβ) plaques and neurofibrillary tangles within the brain parenchyma. It is now well accepted that neuroinflammation constitutes an important feature in AD, wherein the exact role of innate immunity remains unclear. Although innate immune cells are at the forefront to protect the brain in the presence of toxic molecules including Aβ, this natural defense mechanism seems insufficient in AD patients. Monocytes are a key component of the innate immune system and they play multiple roles, such as the removal of debris and dead cells via phagocytosis. These cells respond quickly and mobilize toward the inflamed site, where they proliferate and differentiate into macrophages in response to inflammatory signals. Many studies have underlined the ability of circulating and infiltrating monocytes to clear vascular Aβ microaggregates and parenchymal Aβ deposits respectively, which are very important features of AD. On the other hand, microglia are the resident immune cells of the brain and they play multiple physiological roles, including maintenance of the brain’s microenvironment homeostasis. In the injured brain, activated microglia migrate to the inflamed site, where they remove neurotoxic elements by phagocytosis. However, aged resident microglia are less efficient than their circulating sister immune cells in eliminating Aβ deposits from the brain parenchyma, thus underlining the importance to further investigate the functions of these innate immune cells in AD. The present review summarizes current knowledge on the role of monocytes and microglia in AD and how these cells can be mobilized to prevent and treat the disease.

## Introduction

Alzheimer’s disease (AD) is the most prevalent cause of dementia in older people worldwide. This disease is a neurodegenerative disorder characterized by the progressive loss of memory and cognitive functions. Amyloid-beta (Aβ) deposition in brain parenchyma and blood vessels constitutes a major pathological hallmark of AD [1]. Neurotoxic Aβ_1–40_ and Aβ_1–42_ peptides derived from the sequential proteolytic cleavage of the amyloid precursor protein (APP), mediated by the activity of β-secretases and γ-secretases, accumulate and form soluble oligomers, which over time aggregate to form extracellular insoluble Aβ plaques [1].

Cerebral soluble Aβ accumulation has been proposed to be associated with faulty clearance of this peptide from the brain [2]. The early formation and accumulation of Aβ oligomers in the cerebral vasculature causes the brain’s microvascular dysfunction and contributes to the development of cerebral amyloid angiopathy (CAA), which takes place in 80% of AD cases [3]. Interestingly, microvascular blood–brain barrier (BBB) dysfunction has been reported in early stages of AD [4]. The BBB collaborates with the periphery and brain parenchyma in order to eliminate Aβ from the brain through several sophisticated mechanisms. These mechanisms include Aβ oligomer degradation by specialized enzymes [5], soluble Aβ transport by specialized transport systems [3,6], soluble Aβ elimination via the cerebral interstitial fluid bulk flow [7], soluble Aβ elimination by vascular patrolling monocytes [8] and soluble and insoluble Aβ internalization and degradation by microglia [9].

Although the link between parenchymal Aβ plaque deposition and cognitive decline remains controversial, the detrimental roles of soluble Aβ oligomers in the AD brain have been demonstrated [1], such as inflammation. Aβ-induced inflammation has been shown to be mediated via different mechanisms, including inflammasome activation [10,11], microglia activation [12], reactive astrocytes [13] and monocyte recruitment to brain vasculature, infiltration into brain parenchyma and their subsequent activation [14]. Several studies have demonstrated a close relationship between neuroinflammation and AD pathology [15]. Until recently, neuroinflammation in AD has been exclusively linked to Aβ [16]. However, recent studies have outlined a potential contribution of systemic and local mild chronic inflammation in initiating the neurodegenerative cascade observed in AD [17,18]. Although the link between neuroinflammation and AD pathology is now well recognized, how brain innate immunity is driven in AD is still a matter of debate – especially whether neuroinflammation can be triggered by age-related systemic inflammation [19]. This phenomenon can directly mediate BBB dysfunction in the early stages of AD, thus triggering mild chronic cerebral inflammation that evolves over time [3].

In this review, we aim to highlight the dynamics of monocytes and microglia in AD. More precisely, we will review their interaction with the BBB and brain parenchyma and the implication of such an interaction on AD pathogenesis. Finally, we will be outlining potential approaches that aim to target these cells, such as cell transplantation and immunomodulation, in order to develop novel therapeutic approaches for AD.

## Review

### Monocytes

#### Origin and function

Monocytes constitute a population of circulating leukocytes that are central cells of the innate immune system. They are part of the mononuclear phagocyte system that arises from the hematopoietic system, which is constituted by self-renewal hematopoietic stem cells and progenitor cells located in the bone marrow (BM) [20]. Monocytes come from the monocyte–macrophage dendritic cell progenitor and are incompletely differentiated cells that give rise to a heterogeneous mononuclear phagocyte lineage [20]. They express multiple clusters of differentiation (CD), namely CD115, CD11c, CD14 and CD16 in human or CD115, CD11b and Ly6C in mouse [21]. In parallel, both human and murine monocytes express different levels of chemokine receptors, among which are chemokine (C-X3-C motif) receptor 1 (CX3CR1) and chemokine (C-C motif) receptor 2 (CCR2) [22]. In human, monocytes are regrouped into three main subsets based on their CD14 and CD16 expression levels, which are the classical subset (CD14^++^CD16^−^), the intermediate subset (CD14^++^CD16^+^) and the nonclassical subset (CD14^+^CD16^++^) [23]. In mouse, monocytes are regrouped into two main subsets based on chemokine receptors and Ly6C expression levels; namely the proinflammatory subset (CX3CR1^low^CCR2^+^Ly6C^high^) that is actively recruited to inflamed tissues and contributes to inflammatory responses, and the anti-inflammatory subset (CX3CR1^high^CCR2^−^Ly6C^low^) that constitutes the resident patrolling monocyte population which patrols the lumen of blood vessels and promotes tissue repair [22].

Monocytes are very potent phagocytic cells that respond to stress signals by expressing a variety of surface molecules, among which are scavenger receptors (for example, scavenger receptor SR-A, CD36), low-density lipoprotein receptors (for example, low-density lipoprotein receptor-related protein, LRP1), toll-like receptors (for example, TLR2, TLR4), chemokine receptors (for example, CCR2, CX3CR1), cytokine receptors (for example, macrophage colony-stimulating factor (M-CSF) receptor), Fcγ receptors and adhesion molecules (for example, leukocyte function-associated antigen, LFA-1), wherein the expression level of these molecules reflects their respective functions [21].

**Figure 1:**
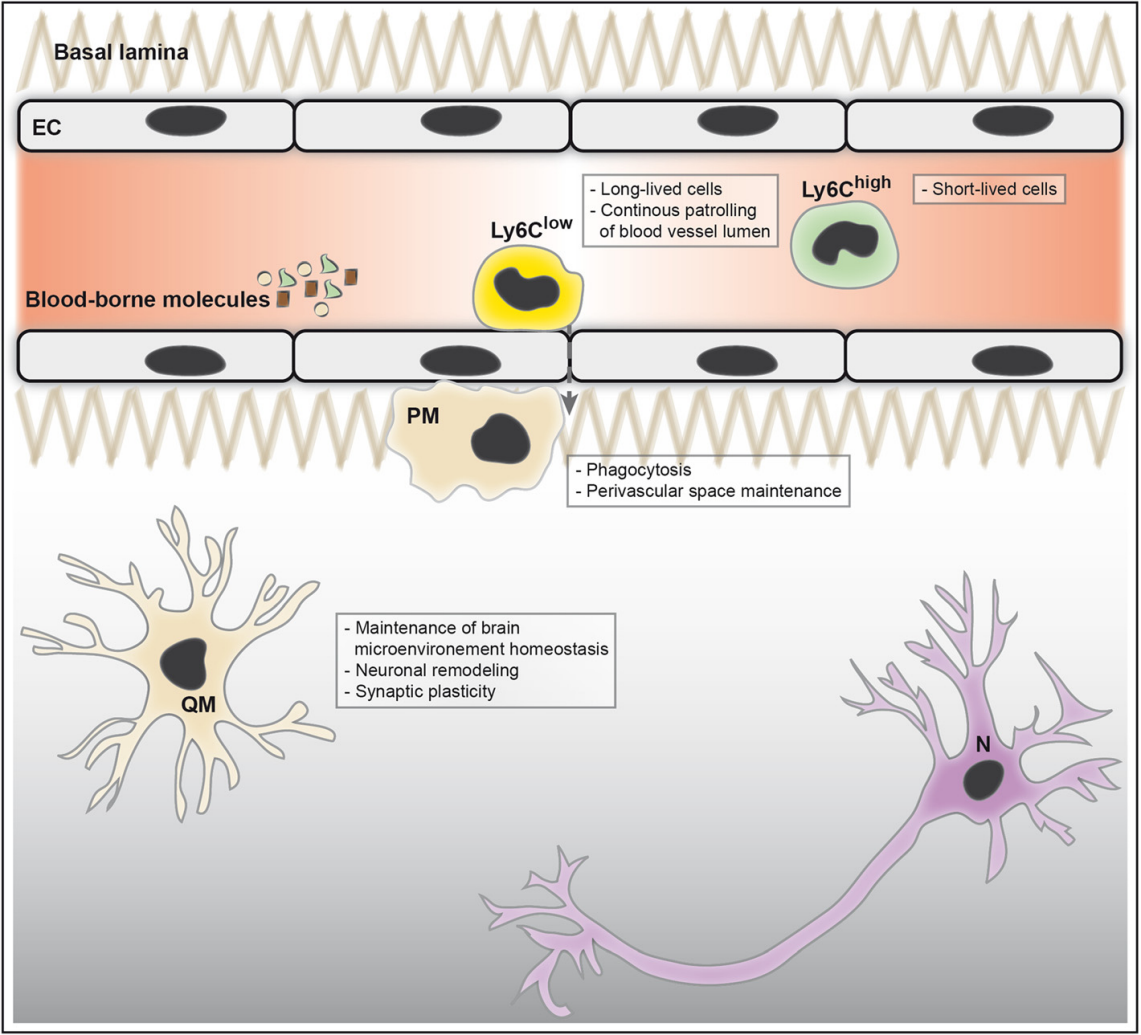
**Innate immunity profile in the healthy brain.** Intact blood–brain barrier (BBB) formed by tightly sealed endothelial cells (EC) and the basal lamina containing extracellular matrix components (for example, collagen, fibronectin). The BBB restricts entry into the brain of pathogens, toxins and blood-borne molecules, such as immunoglobulin, albumin, thrombin, plasmin, fibrin and laminin. Bone marrow-derived circulating monocytes are divided in two main subsets, which are the patrolling anti-inflammatory (Ly6C^low^) monocytes and the circulating proinflammatory (Ly6C^high^) monocytes. Ly6C^low^ monocytes are long-lived cells that ensure continuous surveillance by crawling on blood vessel lumen. Ly6C^high^ monocytes are short-lived cells that are present in blood circulation. Perivascular macrophages (PM) probably arise from Ly6C^low^ monocytes and contribute to the maintenance of homeostasis of the perivascular space, mainly via its phagocytic activity. Quiescent microglia (QM) maintain a healthy brain microenvironment suitable for neurons (N), by continuously sensing any occurring changes via their high ramifications, secreting neurotrophic factors, namely brain-derived neurotrophic factor, and promoting neuronal remodeling and synaptic plasticity.

**Figure 2:**
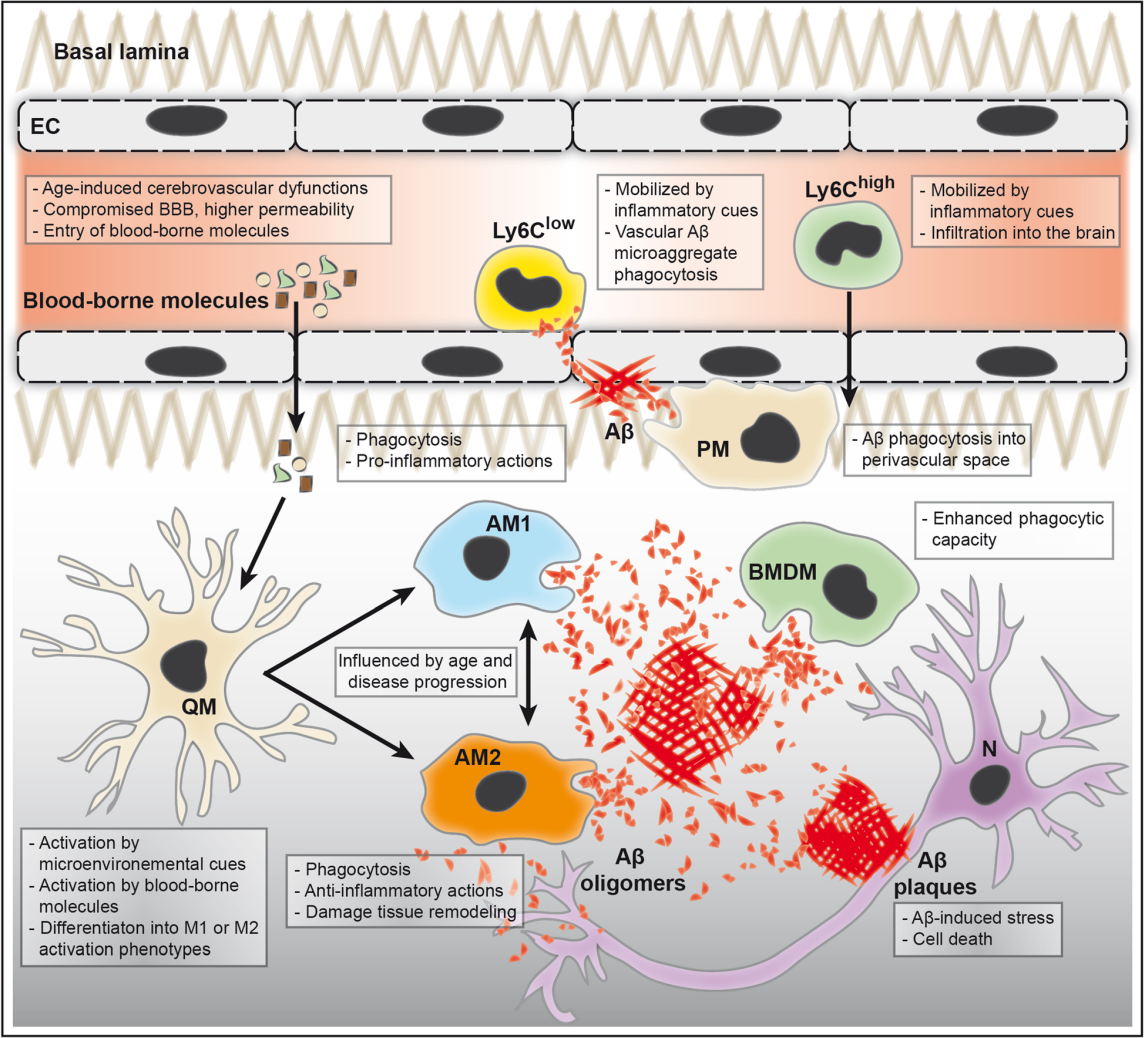
**Innate immune responses in the Alzheimer’s disease brain.** Age-induced cerebrovascular dysfunction induces deregulation of tight junction protein expression, which compromises the integrity of the blood–brain barrier (BBB). A compromised BBB promotes the entry of blood-borne molecules within the perivascular space and brain parenchyma. Patrolling (Ly6C^low^) monocytes are mobilized by inflammatory cues triggered by vascular amyloid-beta (Aβ) microaggregates, contributing to their phagocytosis. Circulating proinflammatory (Ly6C^high^) monocytes are also mobilized by brain-derived inflammatory cues, adhere to brain endothelium and consequently infiltrate brain parenchyma. Aβ-induced inflammatory conditions promote the differentiation of Ly6C^high^ monocytes into bone marrow-derived macrophages (BMDM) that exhibit enhanced Aβ phagocytic activity. Perivascular macrophages (PM) could contribute to parenchymal Aβ deposit elimination via an efficient Aβ species clearance at the BBB. In an Aβ-induced inflammatory microenvironment, neurons (N) become stressed leading to their dysfunction and ultimately their death. Taken together, the presence of Aβ plaques, soluble Aβ species, proinflammatory molecules and blood-borne molecules constitute a stressful microenvironment that activates the quiescent microglia (QM). Amoeboid activated microglial cells can adopt two main phenotypes that coexist in Alzheimer's disease brain: M1 classically activated microglia (AM1) and M2 alternatively activated microglia (AM2). The switch between these two extreme phenotypes is influenced by age and disease progression. The AM1 phenotype is involved in Aβ phagocytosis and proinflammatory actions, such as secretion of cytokines/chemokines within the brain parenchyma. The AM2 phenotype is also involved in Aβ phagocytosis, but in contrast they have anti-inflammatory actions, including damaged tissue repair and remodeling, and cytokine/chemokine production. EC, endothelial cells.

Monocytes are involved in innate immunity by defending the organism against pathogens and toxins [21]. Little is known about monocyte interaction with the brain under physiological conditions. However, it has been proposed that circulating monocytes – more precisely, the patrolling subset that has a long half-life [22] – replenish the perivascular macrophage population in normal tissue, which is involved in maintenance of homeostasis of the perivascular space (Figure 1) [24]. Under pathophysiological conditions, short-lived circulating proinflammatory monocytes are mobilized from the BM to the blood circulation in a CCR2-dependent manner [25,26]. These cells have been shown to possess the capacity to infiltrate inflamed tissues of several organs, including the brain [23]. The infiltration rate of monocytes increases in response to brain-derived inflammatory cues [27]. Following injured brain infiltration, monocytes can differentiate into activated macrophages that are involved in the production of various inflammatory molecules, such as interleukin-1β and tumor necrosis factor α [21], and phagocytosis of toxic elements, including Aβ [27]. It is noteworthy to mention that morphologically these monocyte-derived macrophages are indistinguishable from brain resident microglial cells, but functionally they show a more efficacious phagocytic capacity (Figure 2) [27]. As discussed, the infiltration of monocyte subsets in the inflamed brain and their differentiation into macrophages totally depend on the inflammatory cues present within their microenvironment.

#### Monocyte dynamics in Alzheimer’s disease

##### Monocyte interactions with the blood–brain barrier

Although both monocyte subsets interact with the brain in AD, the anti-inflammatory monocyte subset seems to have a more functionally intimate relationship with the BBB compared with the proinflammatory subset. On the other hand, the interaction of the proinflammatory subset with the BBB is mainly restricted to the process of transmigration, which is an obligatory process to reach brain parenchyma. For instance, it has been shown that anti-inflammatory monocytes behave as housekeepers within the vasculature by surveying the endothelium [28,29]. Several reports outlined the importance of these anti-inflammatory monocytes in AD. More precisely, it has recently been shown that the nonclassical CD14^+^CD16^++^ monocytes in human, which are comparable with mouse anti-inflammatory CX3CR1^high^CCR2^−^Ly6C^low^ monocytes, are reduced in AD patients compared with mild cognitive impairment patients or age-matched healthy controls [30]. In addition, our group demonstrated using the two-photon intravital imaging approach that the patrolling monocyte subset adhered in a specific manner to Aβ-rich brain vasculature, and efficaciously eliminated Aβ microaggregates by internalizing and transporting them from the brain microvasculature to the blood circulation (Figure 2) [8]. BM-derived progenitor cells isolated from Nr4a1^−/−^ mice, which is a transcription factor implicated in the differentiation of anti-inflammatory Ly6C^low^ monocytes within the BM and their survival [31], were transplanted in APP/PS1 mice to address their role in this observation [8]. Importantly, this specific depletion of the anti-inflammatory monocyte subset in APP/PS1 mice increased Aβ deposition within the brain vasculature, which was sufficient to increase overall brain Aβ levels, thus worsening the cognitive function of these mice [8]. Taken together, these observations outline the crucial role of the interaction of these cells with the brain vasculature in AD.

##### Monocyte interactions with the brain parenchyma

Circulating monocytes are able to infiltrate the brain in AD [27]. BM-derived macrophages, which originate essentially from infiltrated proinflammatory monocytes, have been shown to be more efficacious than resident microglia in clearing cerebral Aβ deposits in AD models [9]. Monocyte chemoattractant protein (MCP)-1 (or chemokine (C-C motif) ligand 2 (CCL2)), which is produced by Aβ-induced activated microglial cells, triggers the mobilization of proinflammatory monocytes in the inflamed brain through CCR2 (that is, MCP-1 receptor) (Figure 2) [23]. This MCP-1/CCR2 axis seems to be crucial for monocyte recruitment and infiltration into the brain of APP/PS1 mice, as the depletion of CCR2 reduced the infiltration of these cells in the inflamed brain parenchyma, and consequently reduced the presence of BM-derived macrophages in the vicinity of Aβ plaques, thus increasing cerebral Aβ deposition [32,33]. This observation highlights the role of the MCP-1/CCR2 axis in the recruitment of proinflammatory monocytes into the inflamed brain and their subsequent contribution to parenchymal Aβ clearance. However, it was recently demonstrated that interleukin-1β overexpression in the hippocampus of CCR2-deficient APP/PS1 mice significantly reduced the amyloid plaques loading in the inflamed hippocampus [34]. Interestingly, immune cells were still observed in the hippocampus of these mice, thus suggesting that CCR2^+^ monocytes are not involved in interleukin-1β-mediated Aβ deposit clearance [34]. This observation is highly important because it suggests the implication of other immune cell types that are recruited into the inflamed brain independently of the MCP-1/CCR2 axis. Although infiltrated monocytes are considered more efficacious than resident microglia in Aβ clearance, impaired phagocytic capacity of circulating monocytes has been reported in AD. For instance, Aβ phagocytosis by monocytes isolated from the blood of AD patients showed poor differentiation into macrophages, reduced Aβ internalization and increased apoptosis, comparative with age-matched controls [35]. Recently, an expression quantitative trait locus study performed in purified AD patients’ leukocytes has identified monocyte-specific susceptibility alleles, namely CD33 [36], that are associated with diminished Aβ internalization [37].

In the perivascular space, a distinct population of macrophages exists that is characterized by the expression of acid phosphatase, the activity of nonspecific esterase, the expression of the scavenger receptor CD163 and the expression of mannose receptor CD206 [38]. In contrast to normal resident microglia, perivascular macrophages are regularly replenished by the differentiation of infiltrating monocytes (Figure 1) [39]. Although little is known about perivascular macrophages, they have been demonstrated to act as antigen-presenting cells, to possess a phagocytic activity and to actively respond to brain inflammation [38]. Importantly, the specific depletion of these cells in transgenic AD mouse models highly increased Aβ deposition in the brain microvasculature and consequently in the brain parenchyma [38]. This important observation suggests that these cells could somehow assist the BBB in Aβ clearance. Interestingly, it is proposed that an excessive transport of Aβ species from parenchymal Aβ plaques towards blood circulation contributes to CAA development [40]. In parallel, it has been reported that parenchymal Aβ deposit targeting by immunotherapy approaches could trigger vascular Aβ deposition, thus leading to CAA development [40,41]. Therefore, it would be of great interest to look more closely into the implication of such approaches on the activity of perivascular macrophages, which would outline the lacking link between an efficient parenchymal Aβ elimination and efficient Aβ clearance across the BBB.

### Microglia

#### Origin and function

Microglia are the resident macrophages of the brain, and constitute the main active immune cells in the brain. Although the origin of microglia is still elusive, it is well accepted that these cells arise from myeloid precursors and constitute an ontogenically distinct population of mononuclear phagocytes [42]. As such, microglial cells arise from hematopoietic progenitors in the yolk sac during embryogenesis and are generated in the postnatal stage just after the formation of the BBB [39]. In the adult brain, local self-renewal is sufficient for the maintenance of the microglial population pool [39]. Microglia are therefore physiologically dependent on the colony-stimulating factor 1 receptor signaling that is a key regulator of myeloid lineage cells [42], because its ablation in adult mice results in depletion of 99% of the microglial cell population [43].

Microglia survey the brain and are actively involved in maintaining the brain’s microenvironment by rapidly responding to pathogens and/or damage (Figure 1) [24,44]. Moreover, microglial cells adopt a special phenotype and cellular morphology that is characterized by high ramifications that constitute dynamic and motile sentinels, by which microglia sense any occurring change in their close microenvironment [24,45]. Under physiological conditions, recent reports show that microglia actively contribute to neuronal plasticity and circuit function [46]. More precisely, microglial cells are suggested to be involved in controlling neuronal circuits’ maturation and shaping neuronal connectivity [47]. The chemokine (C-X3-C motif) ligand 1 (CX3CL1; also called fractalkine) signaling pathway plays a key role in this physiological interaction between microglia and neurons [47]. CX3CL1 is secreted by neurons and binds to its receptor, CX3CR1, which is exclusively expressed on microglial cells in the healthy brain [46]. The CX3CL1/CX3CR1 axis plays a crucial role in regulating microglial dynamic surveillance and migration throughout the brain parenchyma, thus ensuring the survival of developing neurons and the maintenance of developing and matures synapses. This axis is therefore directly involved in brain functional connectivity, adult hippocampal neurogenesis and the behavioral outcome [46].

Under pathophysiological conditions, microglial cells are activated and acquire a new morphology characterized by an amoeboid shape. Activated microglial cells are capable of performing several macrophage-like immune functions, such as cytokine release and phagocytosis (Figure 2) [44,45]. In parallel with the newly acquired morphological shape, activated microglia upregulate several key surface markers involved in phagocytosis, namely macrophage antigen complex (Mac)-1 and SR-A [45]. Once activated, microglia can adopt diverse phenotypes ranging between two extremes: a classically activated M1 phenotype that is involved in proinflammatory actions, and an alternatively activated M2 phenotype that is mainly involved in anti-inflammatory actions and tissue repair (Figure 2) [39]. The molecular cues present within the microglial microenvironment play a crucial role in mediating their activation phenotype. It is important to mention that, in the diseased brain tissue, both extremes cohabit within a spectrum of different intermediate phenotypes.

#### Microglia dynamics in Alzheimer’s disease

##### Microglial cell interactions with the blood–brain barrier

The neurovascular unit, which is constituted by endothelial cells, extracellular matrix, pericytes, astrocytes, microglia and neurons, regulates the brain microenvironment by controlling cerebral microcirculation and adjusting the BBB’s parameters based on brain needs [3]. Being a main constituent of the neurovascular unit, microglia are actively involved in maintaining a healthy brain microenvironment that is crucial for neuronal function and survival [48]. In parallel, the activation of microglia is narrowly dependent on their local microenvironment. As mentioned, BBB abnormalities and alterations have been reported in the early stages of AD development [49]. More precisely, it has been suggested that, at the very early stages of the disease, the brain microcirculation is impaired and leads to microvascular dysfunction, thus leading to cerebral chronic hypoperfusion [4]. These early events impair BBB function, leading to a faulty clearance of Aβ oligomers and its accumulation within the brain, which induces neuronal stress [2]. At this stage of the disease, microglial cells through their processes begin to sense neuronal stress [24,44].

Over time, Aβ accumulation within the perivascular space worsens BBB dysfunction caused by a significant decrease in the expression of tight junction proteins between brain endothelial cells, thus increasing BBB permeability to blood-borne molecules such as immunoglobulins, albumin, thrombin, plasmin, fibrin and laminin (Figure 2) [3]. The accumulation of these molecules within the perivascular space exacerbates the microvascular damage and triggers BBB total breakdown [3]. Over time, these molecules trigger microglial cell overactivation (Figure 2). In AD/CAA patients, activated microglial cells that are associated with the BBB express increased protein levels of C3b and Mac-1 [50]. Moreover, it has been shown that the interaction between C3b and CD11b with Aβ is increased in AD/CAA patients [50]. It was suggested that these BBB-associated microglia, via their CD11b receptor, deliver Aβ/C3b complex to brain endothelial cells, thus possibly enhancing Aβ elimination across the BBB [50]. This observation is highly important because it outlines interesting mechanisms, via which the BBB and microglia functionally interact to eliminate brain-derived toxic molecules, such as Aβ, which should be further dissected. Besides, microglial cells have been shown to express high levels of the ATP-binding cassette transporter subfamily A member (ABCA1; that is, cholesterol efflux regulatory protein), which is an efflux pump for cholesterol and phospholipids that contribute to apolipoprotein E lipidation in the brain [51]. The rate of apolipoprotein E lipidation is tightly involved in mediating Aβ uptake by the former, thus contributing to Aβ clearance through the BBB via endothelial LRP1 [52,53]. In parallel, a recent study in APP/PS1 mice showed that the administration of bexarotene, which is a retinoid X receptor agonist, specifically induced apolipoprotein E expression by microglia, which resulted in enhanced clearance of soluble Aβ [54]. Taken together, these observations suggest a highly dynamic and functional interaction at the neurovascular unit, between microglia and the BBB, which has deep implications in Aβ clearance.

##### Microglial activity within the brain parenchyma

In AD, microglia constitute the first responders to cerebral Aβ accumulation, as they have been shown to be highly associated with Aβ plaques and involved in Aβ phagocytosis [9,55]. Microglial cells are directly activated by most Aβ species via several mechanisms that include pattern recognition receptors such as TLRs, and other receptors including receptor for advanced end glycation products (RAGE), LRP1, scavenger receptors and complement receptors [44,48]. Several hypotheses have been formed to explain this distinctive feature of microglia surrounding Aβ plaques. The first initial hypothesis suggested that microglia are exclusively proinflammatory in AD and have a detrimental role in the disease’s development [27,56]. As such, some studies reported the regression of AD pathogenic features following nonsteroidal anti-inflammatory drug treatment [56]. However, clinical trials using nonsteroidal anti-inflammatory drugs to treat AD were inconclusive [56].

The role of microglia in the AD brain was therefore revised, and several recent and emerging data are suggesting a more complex role of microglial cells in AD [15]. As a crucial component associated with microglia’s physiological role, the contribution of the CX3CL1/CX3CR1 axis in AD pathogenesis has been actively investigated. For instance, it has been shown that the ablation of CX3CR1 in AD mouse models, namely APP/PS1 and R1.40, attenuates Aβ deposition by modulating the phagocytic activity of microglial cells [57]. By contrast, a study performed in the 5 × Tg-AD mouse model revealed that CX3CR1-deficient microglia did not affect Aβ levels, but prevent neuronal loss [58]. These observations therefore highlight important concerns about experimental parameters, such as transgenic animal models and neuroinflammatory conditions, which impact differently on the CX3CR1 signaling involved in neuron–microglia communication. In parallel, the efficacy of resident microglia that surround Aβ plaques in degrading Aβ species is still elusive. As such, microglia that are spatially associated with Aβ plaques have been shown to contain Aβ species in their endoplasmic reticulum, a nonphagocytic specialized organelle, suggesting that resident microglia do not actively participate in Aβ phagocytosis [59]. By contrast, it has been shown that microglia are indeed capable of internalizing fibrillar and soluble Aβ, but are unable to process these peptides [60]. Importantly, in AD patients that underwent a cerebral ischemic attack, which highly compromised the BBB, circulating monocytes massively infiltrate the brain parenchyma where they differentiate into macrophages [61]. These infiltrated macrophages contained Aβ species within their lysosomes, a specialized phagocytic organelle, pointing toward an efficacious phagocytosis [61]. Moreover, it has been shown that APP/PS1 mice irradiation and subsequent transplantation of BM-derived progenitor cells gave rise to monocyte-derived microglial cells, which originate from infiltrating monocytes capable of migrating throughout brain parenchyma, specifically surround Aβ plaques and efficaciously eliminate the latter (Figure 2) [9]. Taken together, these observations suggest a crucial impact of brain parenchyma microenvironment on cells’ phagocytic capacity. For instance, newly infiltrated macrophages, which were less exposed to Aβ aggregates and proinflammatory cues, appear more efficient than brain resident microglia, which were highly exposed to Aβ aggregates and proinflammatory cues.

AD is an age-related progressive neurodegenerative disease with different development stages, which could explain the multifaceted roles of microglia in AD. Microglial cells undergo significant changes in their phenotype, and their activity is impaired with age. In aged brain, microglial cells exhibit an altered shape and dystrophic processes, and seem to be hyper-responsive to mild inflammatory stimulations [62]. Importantly, most proinflammatory cytokines that are produced by aged microglia are controlled by the CX3CL1/CX3CR1 signaling pathway [63], which translates a progressive dysfunctional interaction between microglia and neurons with age. In AD, the early activation of microglial cells has been proposed to be beneficial by promoting clearance of Aβ before plaque formation [64]. However, over time microglial cells lose their protective role, due to the persistent production and accumulation of proinflammatory cytokines within their microenvironment [65]. Under such conditions, microglial cells become hypersensitive and play a detrimental role through the excessive continuous production and secretion of proinflammatory and neurotoxic molecules [65]. In parallel, the expression levels of several microglial markers involved in Aβ uptake and phagocytosis have been shown to be impaired [65]. Interestingly, RNA sequencing in aged microglia has identified numerous age-related microglial changes, such as a downregulation of transcripts encoding for endogenous ligand recognition proteins, an upregulation of those involved in host defense and pathogen recognition, in addition to an increased expression of neuroprotective genes [66]. This observation is interesting because it suggests that microglia can adopt a neuroprotective phenotype with age. Therefore, it is important to take these factors into consideration when drawing a complete picture of the role of microglia in AD pathogenesis.

### Targeting monocytes and microglia as a novel therapeutic approach in Alzheimer’s disease

Monocytes and microglia constitute two major players involved in AD etiology. Lessons obtained from many recent studies highlighted these cells as potential targets for AD treatment.

#### Cell therapy

Several studies have shown that progenitor cell transplantation decelerates the pathogenic features of AD by affecting mainly brain innate immune function. An elegant study reported that the systemic administration of human umbilical cord blood cells reduced the levels of parenchymal and vascular Aβ by specifically increasing the phagocytic capacity of microglial cells and by inhibiting interferon γ mediated microglial activation [67]. Interestingly, it has been suggested that the monocytes derived from healthy individuals phagocyte Aβ more efficiently than monocytes derived from AD individuals [68]. In parallel, as mentioned, our group has shown that microglial cells which originate from BM-derived progenitor cells are more efficacious in Aβ phagocytosis and clearance compared with resident microglia [9]. Taken together, these observations are extremely important because they outline the transplantation of BM-derived progenitor cells from healthy individuals into AD individuals as a potential therapeutic approach. Indeed, it has been shown that the intracerebral transplantation of BM-derived mesenchymal stem cells reduced Aβ deposition and enhanced the cognitive functions of an AD mouse model, mainly by modulating brain immune responses [69]. Recently, the transplantation of adipose-derived mesenchymal stem cells, which are considered as a new cell source for regenerative therapy, has been shown to be a promising avenue in treating AD [70]. The transplantation of these cells decelerates the pathogenic features of AD in a mouse model of AD by alternatively activating microglial cells, which was translated by the cells’ reduced production of proinflammatory mediators and accompanied by an increased expression of microglial-derived enzymes involved in Aβ degradation [70].

Interestingly, the beneficial effects of stem/progenitor cell transplantation seem to go beyond the cell’s capacity to directly differentiate into microglial cells. More precisely, stem/progenitor cell transplantation has been proposed to also modulate the microenvironment of resident microglial cells and to enhance the metabolic activity in the vicinity of microglia. For example, an *in vitro* study showed that the co-culture of the immortalized murine microglial cell line BV2 with human umbilical cord blood-derived mesenchymal stem cells increased the microglial cell expression of neprilysin, an enzyme involved in Aβ degradation [71]. The transplantation of these cells in an AD mouse model reduced Aβ deposition, which was neprilysin dependent [71].

#### Cell stimulation and immunomodulation

As mentioned, resident microglial cells surrounding Aβ plaques are not efficacious in degrading Aβ. Nonetheless, it has been shown that their stimulation could enhance their intrinsic phagocytic capacity to degrade Aβ more efficaciously. Moreover, it has been proposed that a shift from a classical activation M1 phenotype that exacerbates the inflammatory response towards an alternative activation M2 phenotype that promotes tissue repair would enhance cerebral Aβ clearance [11].

As such, an early study showed beneficial effects of an intra-hippocampal injection of lipopolysaccharide, which is a TLR4 ligand, in a mouse model of AD [72]. The authors observed an increased activation of resident microglial cells, which was accompanied by a significant reduction of cerebral Aβ load within the brain parenchyma of mice following lipopolysaccharide administration [72]. These results outline that the early activation of microglia promotes Aβ phagocytosis, while later activation could contribute to chronic inflammation and neurodegeneration. In parallel, our group recently demonstrated that the chronic systemic administration of a detoxified TLR4 ligand, which is a lipopolysaccharide derivative called monophosphoryl lipid A, potently decelerated AD-related pathology in a mouse model of AD, by significantly reducing cerebral Aβ deposits and ameliorating the cognitive functions of these mice [73]. Monophosphoryl lipid A early treatment enhanced Aβ phagocytosis by monocytes and microglia without inducing a potentially harmful inflammatory response, such as observed with lipopolysaccharide.

Other similar strategies using molecules that modulate monocyte and microglial activity have also showed interesting results. M-CSF is a hematopoietic growth factor involved in the proliferation, differentiation and survival of monocytes, macrophages and BM-derived progenitor cells [74]. M-CSF receptor overexpression in an AD mouse model resulted in an increased antibody-opsonized Aβ phagocytosis by microglial cells [75]. In parallel, M-CSF treatment of a mouse model of AD improved their cognitive function, which was accompanied by reduced Aβ deposits in brain parenchyma [76]. Importantly, M-CSF treatment increased the number of microglial cells surrounding plaques, which was accompanied by a higher rate of Aβ internalization by these cells [76]. Taken together, these observations showed that the early activation of monocytes and microglia constitutes an interesting strategy to, at least, decelerate AD progression. Moreover, these studies underlie the beneficial roles of such molecules as modulator of immune responses, which potentiate the intrinsic phagocytic capacity of monocytes and microglia without triggering an exacerbated inflammation that could worsen AD pathology.

Finally, the lipid mediator palmitoylethanolamide, which is an endogenous fatty acid amide present in microglial cells, has been reported to modulate the microglial cell phenotype [77]. Indeed, palmitoylethanolamide has been suggested to be involved in controlling microglial cell alternative activation by enhancing their migration capacity, via its interaction with a cannabinoid-like receptor [77]. Interestingly, a recent study reported an unknown therapeutic potential of palmitoylethanolamide in AD. More precisely, in wildtype mice that were intracerebrally injected with Aβ peptides, the administration of palmitoylethanolamide dose-dependently reduced Aβ-induced memory impairments in a peroxisome proliferator-activated receptor alpha-dependent manner [78].

## Conclusions

In this review, we have attempted to underline the role of monocytes and microglia in AD. Moreover, we outlined their relevance for the development of novel therapeutic strategies. The role of neuroinflammation in AD is still a matter of debate. Many studies have shown conflicting results about the beneficial and deleterious effects of neuroinflammation [15]. However, it is now well accepted that there is no ultimately good or bad neuroinflammation; it is context dependent. On one hand, neuroinflammation mediates neuroprotective effects by forming the first line of defense in the brain; on the other, it mediates neurotoxic effects by exacerbating the inflammatory response. Monocytes and microglia are key innate immune cells implicated in AD etiology. However, it is now urgent to further investigate the multifaceted roles of these cells in AD by outlining the complex regulatory molecular mechanisms that govern the balance between their beneficial and detrimental effects in a context-dependent manner, especially during the different stages of the disease’s development and age. Such an approach would allow the development of novel therapeutic strategies that mainly focus on enhancing Aβ elimination, without generating undesirable effects, such as an exacerbated inflammation and neurotoxicity.

## Note:

This article is part of a series on *Innate Immunity*, edited by Donna Wilcock. Other articles in this series can be found at http://alres.com/series/innateimmunity

## Abbreviations

AD: Alzheimer’s disease
APP: Amyloid precursor protein
Aβ: Amyloid-beta
BBB: Blood–brain barrier
BM: Bone marrow
CAA: Cerebral amyloid angiopathy
CCR2: Chemokine (C-C motif) receptor 2
CD: Cluster of differentiation
CX3CL1: Chemokine (C-X3-C motif) ligand 1
CX3CR1: Chemokine (C-X3-C motif) receptor 1
Mac: Macrophage antigen complex
MCP: Monocyte chemoattractant protein
M-CSF: Macrophage colony-stimulating factor
TLR: Toll-like receptor

## References

[1] Selkoe DJ (2012). Preventing Alzheimer's disease. Science..

[2] Sagare AP, Bell RD, Zlokovic BV. Neurovascular dysfunction and faulty amyloid β-peptide clearance in Alzheimer disease. Cold Spring Harb Perspect Med. 2012;2. 10.1101/cshperspect.a011452 PMC3475405 23028132

[3] Zlokovic BV (2011). Neurovascular pathways to neurodegeneration in Alzheimer's disease and other disorders. Nat Rev Neurosci..

[4] Pimentel-Coelho PM, Rivest S (2012). The early contribution of cerebrovascular factors to the pathogenesis of Alzheimer's disease. Eur J Neurosci..

[5] Farris W, Mansourian S, Chang Y, Lindsley L, Eckman EA, Frosch MP (2003). Insulin-degrading enzyme regulates the levels of insulin, amyloid beta-protein, and the beta-amyloid precursor protein intracellular domain in vivo. Proc Natl Acad Sci U S A..

[6] Sagare A, Deane R, Bell RD, Johnson B, Hamm K, Pendu R (2007). Clearance of amyloid-beta by circulating lipoprotein receptors. Nat Med..

[7] Iliff JJ, Wang M, Liao Y, Plogg BA, Peng W, Gundersen GA (2012). A paravascular pathway facilitates CSF flow through the brain parenchyma and the clearance of interstitial solutes, including amyloid β. Sci Transl Med.

[8] Michaud J-P, Bellavance M-A, Préfontaine P, Rivest S (2013). Real-time in vivo imaging reveals the ability of monocytes to clear vascular amyloid beta. Cell Rep..

[9] Simard AR, Soulet D, Gowing G, Julien J-P, Rivest S (2006). Bone marrow-derived microglia play a critical role in restricting senile plaque formation in Alzheimer's disease. Neuron..

[10] Halle A, Hornung V, Petzold GC, Stewart CR, Monks BG, Reinheckel T (2008). The NALP3 inflammasome is involved in the innate immune response to amyloid-β. Nat Immunol..

[11] Heneka MT, Kummer MP, Stutz A, Delekate A, Schwartz S, Vieira-Saecker A (2013). NLRP3 is activated in Alzheimer’s disease and contributes to pathology in APP/PS1 mice. Nature..

[12] Bornemann KD, Wiederhold K-H, Pauli C, Ermini F, Stalder M, Schnell L (2001). Aβ-induced inflammatory processes in microglia cells of APP23 transgenic mice. Am J Pathol..

[13] White JA, Manelli AM, Holmberg KH, Van Eldik LJ, LaDu MJ (2005). Differential effects of oligomeric and fibrillar amyloid-β1–42 on astrocyte-mediated inflammation. Neurobiol Dis..

[14] Gonzalez-Velasquez FJ, Moss MA (2008). Soluble aggregates of the amyloid-beta protein activate endothelial monolayers for adhesion and subsequent transmigration of monocyte cells. J Neurochem..

[15] Wyss-Coray T, Rogers J (2012). Inflammation in Alzheimer disease – a brief review of the basic science and clinical literature. Cold Spring Harb Perspect Med..

[16] Akiyama H, Barger S, Barnum S, Bradt B, Bauer J, Cole GM (2000). Inflammation and Alzheimer's disease. Neurobiol Aging..

[17] Zhang R, Miller RG, Madison C, Jin X, Honrada R, Harris W (2013). Systemic immune system alterations in early stages of Alzheimer's disease. J Neuroimmunol..

[18] Cunningham C (2012). Microglia and neurodegeneration: the role of systemic inflammation. Glia..

[19] Pimplikar SW (2014). Neuroinflammation in Alzheimer's disease: from pathogenesis to a therapeutic target. J Clin Immunol..

[20] Geissmann F, Manz MG, Jung S, Sieweke MH, Merad M, Ley K (2010). Development of monocytes, macrophages, and dendritic cells. Science..

[21] Ginhoux F, Jung S (2014). Monocytes and macrophages: developmental pathways and tissue homeostasis. Nat Rev Immunol..

[22] Auffray C, Sieweke MH, Geissmann F (2009). Blood monocytes: development, heterogeneity, and relationship with dendritic cells. Annu Rev Immunol..

[23] Naert G, Rivest S (2013). A deficiency in CCR2+ monocytes: the hidden side of Alzheimer's disease. J Mol Cell Biol..

[24] Hanisch U-K, Kettenmann H (2007). Microglia: active sensor and versatile effector cells in the normal and pathologic brain. Nat Neurosci..

[25] Mildner A, Mack M, Schmidt H, Bruck W, Djukic M, Zabel MD (2009). CCR2 + Ly-6Chi monocytes are crucial for the effector phase of autoimmunity in the central nervous system. Brain..

[26] Tsou C-L, Peters W, Si Y, Slaymaker S, Aslanian AM, Weisberg SP (2007). Critical roles for CCR2 and MCP-3 in monocyte mobilization from bone marrow and recruitment to inflammatory sites. J Clin Invest..

[27] Malm T, Koistinaho M, Muona A, Magga J, Koistinaho J (2010). The role and therapeutic potential of monocytic cells in Alzheimer's disease. Glia..

[28] Carlin LM, Stamatiades EG, Auffray C, Hanna RN, Glover L, Vizcay-Barrena G (2013). Nr4a1-dependent Ly6Clow monocytes monitor endothelial cells and orchestrate their disposal. Cell..

[29] Auffray C, Fogg D, Garfa M, Elain G, Join-Lambert O, Kayal S (2007). Monitoring of blood vessels and tissues by a population of monocytes with patrolling behavior. Science..

[30] Saresella M, Marventano I, Calabrese E, Piancone F, Rainone V, Gatti A (2014). A complex proinflammatory role for peripheral monocytes in Alzheimer's disease. J Alzheimers Dis..

[31] Hanna RN, Carlin LM, Hubbeling HG, Nackiewicz D, Green AM, Punt JA (2011). The transcription factor NR4A1 (Nur77) controls bone marrow differentiation and the survival of Ly6C-monocytes. Nat Immunol..

[32] El Khoury J, Toft M, Hickman SE, Means TK, Terada K, Geula C (2007). Ccr2 deficiency impairs microglial accumulation and accelerates progression of Alzheimer-like disease. Nat Med..

[33] Naert G, Rivest S (2011). CC chemokine receptor 2 deficiency aggravates cognitive impairments and amyloid pathology in a transgenic mouse model of Alzheimer's disease. J Neurosci..

[34] Rivera-Escalera F, Matousek SB, Ghosh S, Olschowka JA, O’Banion MK (2014). Interleukin-1β mediated amyloid plaque clearance is independent of CCR2 signaling in the APP/PS1 mouse model of Alzheimer's disease. Neurobiol Dis..

[35] Fiala M, Lin J, Ringman J, Kermani-Arab V, Tsao G, Patel A (2005). Ineffective phagocytosis of amyloid-beta by macrophages of Alzheimer's disease patients. J Alzheimers Dis..

[36] Raj T, Rothamel K, Mostafavi S, Ye C, Lee MN, Replogle JM (2014). Polarization of the effects of autoimmune and neurodegenerative risk alleles in leukocytes. Science.

[37] Bradshaw EM, Chibnik LB, Keenan BT, Ottoboni L, Raj T, Tang A (2013). CD33 Alzheimer's disease locus: altered monocyte function and amyloid biology. Nat Neurosci..

[38] Hawkes CA, McLaurin J (2009). Selective targeting of perivascular macrophages for clearance of β-amyloid in cerebral amyloid angiopathy. Proc Natl Acad Sci U S A..

[39] Prinz M, Priller J (2014). Microglia and brain macrophages in the molecular age: from origin to neuropsychiatric disease. Nat Rev Neurosci..

[40] Weller RO, Preston SD, Subash M, Carare RO (2009). Cerebral amyloid angiopathy in the aetiology and immunotherapy of Alzheimer disease. Alzheimers Res Ther..

[41] Boche D, Zotova E, Weller RO, Love S, Neal JW, Pickering RM (2008). Consequence of Abeta immunization on the vasculature of human Alzheimer's disease brain. Brain..

[42] Ginhoux F, Greter M, Leboeuf M, Nandi S, See P, Gokhan S (2010). Fate mapping analysis reveals that adult microglia derive from primitive macrophages. Science..

[43] Elmore MRP, Najafi AR, Koike MA, Dagher NN, Spangenberg EE, Rice RA (2014). Colony-stimulating factor 1 receptor signaling is necessary for microglia viability, unmasking a microglia progenitor cell in the adult brain. Neuron..

[44] Block ML, Zecca L, Hong J-S (2007). Microglia-mediated neurotoxicity: uncovering the molecular mechanisms. Nat Rev Neurosci..

[45] Saijo K, Glass CK (2011). Microglial cell origin and phenotypes in health and disease. Nat Rev Immunol..

[46] Paolicelli RC, Bisht K, Tremblay M-È (2014). Fractalkine regulation of microglial physiology and consequences on the brain and behavior. Front Cell Neurosci..

[47] Paolicelli RC, Bolasco G, Pagani F, Maggi L, Scianni M, Panzanelli P (2011). Synaptic pruning by microglia is necessary for normal brain development. Science..

[48] Lampron A, ElAli A, Rivest S (2013). Innate immunity in the CNS: redefining the relationship between the CNS and its environment. Neuron..

[49] Bell RD, Zlokovic BV (2009). Neurovascular mechanisms and blood–brain barrier disorder in Alzheimer's disease. Acta Neuropathol..

[50] Zabel M, Schrag M, Crofton A, Tung S, Beaufond P, Van Ornam J (2013). A shift in microglial β-amyloid binding in Alzheimer's disease is associated with cerebral amyloid angiopathy. Brain Pathol..

[51] Hirsch-Reinshagen V, Zhou S, Burgess BL, Bernier L, McIsaac SA, Chan JY (2004). Deficiency of ABCA1 impairs apolipoprotein E metabolism in brain. J Biol Chem..

[52] Wahrle SE, Jiang H, Parsadanian M, Hartman RE, Bales KR, Paul SM (2005). Deletion of Abca1 increases Abeta deposition in the PDAPP transgenic mouse model of Alzheimer disease. J Biol Chem..

[53] Elali A, Rivest S (2013). The role of ABCB1 and ABCA1 in beta-amyloid clearance at the neurovascular unit in Alzheimer's disease. Front Physiol..

[54] Cramer PE, Cirrito JR, Wesson DW, Lee CYD, Karlo JC, Zinn AE (2012). ApoE-directed therapeutics rapidly clear β-amyloid and reverse deficits in AD mouse models. Science..

[55] Malm TM, Koistinaho M, Pärepalo M, Vatanen T, Ooka A, Karlsson S (2005). Bone-marrow-derived cells contribute to the recruitment of microglial cells in response to β-amyloid deposition in APP/PS1 double transgenic Alzheimer mice. Neurobiol Dis..

[56] McGeer PL, McGeer EG (2007). NSAIDs and Alzheimer disease: epidemiological, animal model and clinical studies. Neurobiol Aging..

[57] Lee S, Varvel NH, Konerth ME, Xu G, Cardona AE, Ransohoff RM (2010). CX3CR1 deficiency alters microglial activation and reduces beta-amyloid deposition in two Alzheimer's disease mouse models. Am J Pathol..

[58] Fuhrmann M, Bittner T, Jung CKE, Burgold S, Page RM, Mitteregger G (2010). Microglial Cx3cr1 knockout prevents neuron loss in a mouse model of Alzheimer's disease. Nat Neurosci..

[59] Frackowiak J, Wisniewski HM, Wegiel J, Merz GS, Iqbal K, Wang KC (1992). Ultrastructure of the microglia that phagocytose amyloid and the microglia that produce beta-amyloid fibrils. Acta Neuropathol..

[60] Chung H, Brazil MI, Soe TT, Maxfield FR (1999). Uptake, degradation, and release of fibrillar and soluble forms of Alzheimer's amyloid beta-peptide by microglial cells. J Biol Chem..

[61] Wisniewski HM, Barcikowska M, Kida E (1991). Phagocytosis of beta/A4 amyloid fibrils of the neuritic neocortical plaques. Acta Neuropathol..

[62] Njie EG, Boelen E, Stassen FR, Steinbusch HWM, Borchelt DR, Streit WJ (2012). Ex vivo cultures of microglia from young and aged rodent brain reveal age-related changes in microglial function. Neurobiol Aging.

[63] Limatola C, Ransohoff RM (2014). Modulating neurotoxicity through CX3CL1/CX3CR1 signaling. Front Cell Neurosci..

[64] Krabbe G, Halle A, Matyash V, Rinnenthal JL, Eom GD, Bernhardt U (2013). Functional impairment of microglia coincides with Beta-amyloid deposition in mice with Alzheimer-like pathology. PLoS One..

[65] Hickman SE, Allison EK, Khoury EJ (2008). Microglial dysfunction and defective beta-amyloid clearance pathways in aging Alzheimer's disease mice. J Neurosci..

[66] Hickman SE, Kingery ND, Ohsumi TK, Borowsky ML, Wang L-C, Means TK (2013). The microglial sensome revealed by direct RNA sequencing. Nat Neurosci..

[67] Nikolic WV, Hou H, Town T, Zhu Y, Giunta B, Sanberg CD (2008). Peripherally administered human umbilical cord blood cells reduce parenchymal and vascular beta-amyloid deposits in Alzheimer mice. Stem Cells Dev..

[68] Avagyan H, Goldenson B, Tse E, Masoumi A, Porter V, Wiedau-Pazos M (2009). Immune blood biomarkers of Alzheimer disease patients. J Neuroimmunol.

[69] Lee JK, Jin HK, Endo S, Schuchman EH, Carter JE, Bae J-S (2010). Intracerebral transplantation of bone marrow-derived mesenchymal stem cells reduces amyloid-beta deposition and rescues memory deficits in Alzheimer's disease mice by modulation of immune responses. Stem Cells..

[70] Ma T, Gong K, Ao Q, Yan Y, Song B, Huang H (2013). Intracerebral transplantation of adipose-derived mesenchymal stem cells alternatively activates microglia and ameliorates neuropathological deficits in Alzheimer's disease mice. Cell Transplant..

[71] Kim J-Y, Kim DH, Kim JH, Lee D, Jeon HB, Kwon S-J (2012). Soluble intracellular adhesion molecule-1 secreted by human umbilical cord blood-derived mesenchymal stem cell reduces amyloid-β plaques. Cell Death Differ..

[72] DiCarlo G, Wilcock D, Henderson D, Gordon M, Morgan D (2001). Intrahippocampal LPS injections reduce Abeta load in APP + PS1 transgenic mice. Neurobiol Aging..

[73] Michaud J-P, Hallé M, Lampron A, Thériault P, Préfontaine P, Filali M (2013). Toll-like receptor 4 stimulation with the detoxified ligand monophosphoryl lipid A improves Alzheimer's disease-related pathology. Proc Natl Acad Sci U S A..

[74] Hamilton JA, Achuthan A (2013). Colony stimulating factors and myeloid cell biology in health and disease. Trends Immunol..

[75] Mitrasinovic OM, Murphy GM (2003). Microglial overexpression of the M-CSF receptor augments phagocytosis of opsonized Aβ. Neurobiol Aging..

[76] Boissonneault V, Filali M, Lessard M, Relton J, Wong G, Rivest S (2009). Powerful beneficial effects of macrophage colony-stimulating factor on beta-amyloid deposition and cognitive impairment in Alzheimer's disease. Brain..

[77] Nau R, Ribes S, Djukic M, Eiffert H (2014). Strategies to increase the activity of microglia as efficient protectors of the brain against infections. Front Cell Neurosci..

[78] D'Agostino G, Russo R, Avagliano C, Cristiano C, Meli R, Calignano A (2012). Palmitoylethanolamide protects against the amyloid-β25-35-induced learning and memory impairment in mice, an experimental model of Alzheimer disease. Neuropsychopharmacology..

